# TgTKL1 Is a Unique Plant-Like Nuclear Kinase That Plays an Essential Role in Acute Toxoplasmosis

**DOI:** 10.1128/mBio.00301-18

**Published:** 2018-03-20

**Authors:** Joseph M. Varberg, Isabelle Coppens, Gustavo Arrizabalaga, Rajshekhar Y. Gaji

**Affiliations:** a Department of Pharmacology and Toxicology, Indiana University School of Medicine, Indianapolis, Indiana, USA; b Department of Molecular Microbiology and Immunology, Johns Hopkins Bloomberg School of Public Health, Baltimore, Maryland, USA; University of Pittsburgh

**Keywords:** apicomplexan parasites, kinase, *Toxoplasma gondii*, host cell invasion

## Abstract

In the protozoan parasite *Toxoplasma gondii*, protein kinases have been shown to play key roles in regulating parasite motility, invasion, replication, egress, and survival within the host. The tyrosine kinase-like (TKL) family of proteins are an unexplored set of kinases in *Toxoplasma*. Of the eight annotated TKLs in the *Toxoplasma* genome, a recent genome-wide loss-of-function screen showed that six are important for tachyzoite fitness. By utilizing an endogenous tagging approach, we showed that these six *T. gondii* TKLs (TgTKLs) localize to various subcellular compartments, including the nucleus, the cytosol, the inner membrane complex, and the Golgi apparatus. To gain insight into the function of TKLs in *Toxoplasma*, we first characterized TgTKL1, which contains the plant-like enhanced disease resistance 1 (EDR1) domain and localizes to the nucleus. TgTKL1 knockout parasites displayed significant defects in progression through the lytic cycle; we show that the defects were due to specific impairment of host cell attachment. Transcriptomics analysis identified over 200 genes of diverse functions that were differentially expressed in TgTKL1 knockout parasites. Importantly, numerous genes implicated in host cell attachment and invasion were among those most significantly downregulated, resulting in defects in microneme secretion and processing. Significantly, all of the mice inoculated intraperitoneally with TgTKL1 knockout parasites survived the infection, suggesting that TgTKL1 plays an essential role in acute toxoplasmosis. Together, these findings suggest that TgTKL1 mediates a signaling pathway that regulates the expression of multiple factors required for parasite virulence, underscoring the potential of this kinase as a novel therapeutic target.

## IMPORTANCE

*Toxoplasma gondii* is a protozoan parasite that can cause chronic and life-threatening disease in mammals; new drugs are greatly needed for treatment. One attractive group of drug targets consists of parasite kinases containing unique features that distinguish them from host proteins. In this report, we identify and characterize a previously unstudied kinase, TgTKL1, that localizes to the nucleus and contains a domain architecture unique to plants and protozoa. By disrupting TgTKL1, we showed that this kinase is required for the proper expression of hundreds of genes, including many that are required for the parasite to gain entry into the host cell. Specifically, parasites lacking TgTKL1 have defects in host cell attachment, resulting in impaired growth *in vitro* and a complete loss of virulence in mice. This report provides insight into the importance of the parasite tyrosine kinase-like kinases and establishes TgTKL1 as a novel and essential virulence factor in *Toxoplasma*.

## INTRODUCTION

*Toxoplasma gondii* is an obligate intracellular protozoan that has a very broad intermediate-host range and infects approximately one-third of the human population (1) Following infection with *Toxoplasma*, the acute form of the parasite (tachyzoite) disseminates quickly into various organs in the host. In response to the subsequent host immune response, the parasite evades the immune system by converting into an encysted form (the bradyzoite) and establishing a chronic infection that persists through the life of the host (2). In immunocompromised individuals, such as those infected with HIV, blood cancer patients, and those undergoing immunosuppressive therapy during organ transplantation, rupture of existing bradyzoite cysts or the establishment of new acute infections can lead to fatal toxoplasmosis (3–6). Additionally, transmission from the mother to the fetus during pregnancy results in congenital toxoplasmosis, causing severe disease in the offspring, including neurological deficiencies and blindness, frequently resulting in neonatal death (7). While the available current therapy is effective against the acute stage of infection, it can have toxic side effects and, importantly, does not treat the chronic form of the disease (8, 9). As a result, there is a strong need for the identification and development of novel therapeutic options to treat *Toxoplasma* infections.

The intracellular lifestyle of *Toxoplasma* begins with the parasite gaining entry into host cell through an active invasion mechanism (10). Once in the host cell, the parasite is surrounded by a parasitophorous vacuole (PV) membrane within which the parasite replicates through a process known as endodyogeny (11–13). After undergoing multiple rounds of division, the parasites egress from the host cell, resulting in its destruction (14, 15). Importantly, much of the pathology associated with *Toxoplasma* infection is due to the tissue destruction caused by repeated cycles of invasion, replication, and egress within the infected host (15, 16). Consequently, identification of the unique parasite factors that are required for the invasion, replication, and egress processes is critical for the development of novel therapeutics against toxoplasmosis (15, 16).

Protein kinases are a family of proteins that regulate nearly all biological processes in eukaryotic cells and have been exploited as drug targets in many disease contexts (17). In *Toxoplasma*, kinases have been shown to play a key role in parasite motility, invasion, replication, and egress processes and to promote parasite survival within the host by nullifying host defense mechanisms (18–25). In total, the *Toxoplasma* genome contains 159 putative kinases, of which 108 are predicted to be catalytically active (26). Members of seven of the nine groups of eukaryotic protein kinases (ePK) (26, 27) are present; however, *Toxoplasma* lacks members of the receptor guanylate cyclase (RGC) and tyrosine kinase (TK) groups. In addition to these standard ePKs, *Toxoplasma* contains an expanded group of coccidian-specific secreted rhoptry kinases (ROPK) that includes important virulence factors (22, 26, 28–31). Functional characterization of *Toxoplasma* kinases has shown that many of these proteins have potential as drug targets, and identification and characterization of novel inhibitors of parasite kinases are active areas of research (32–34); however, while representatives of many of the ePK groups have been functionally characterized in *Toxoplasma*, members of the tyrosine kinase-like (TKL) kinase group have remained completely unstudied.

In this report, we provide the first characterization of TKL kinases in *Toxoplasma*. We show that the six putative TKLs that have been suggested to be important for *Toxoplasma* growth *in vitro* localize to multiple, distinct compartments in the parasite. Furthermore, functional characterization of *T. gondii* TKL (TgTKL), a novel plant-like nuclear kinase, revealed a role for this protein in regulating the expression of over 200 genes. The loss of TgTKL1 results in downregulation of multiple invasion-related factors, leading to defects in the secretion and processing of micronemal proteins. This causes significant impairment of parasite growth *in vitro* and a complete loss of virulence *in vivo*. These results establish TgTKL1 as a novel parasite virulence factor and a promising candidate for further exploration as a novel drug target in *Toxoplasma*.

## RESULTS

### Tyrosine kinase-like proteins localize to multiple compartments in *Toxoplasma.*

**FIG 1:**
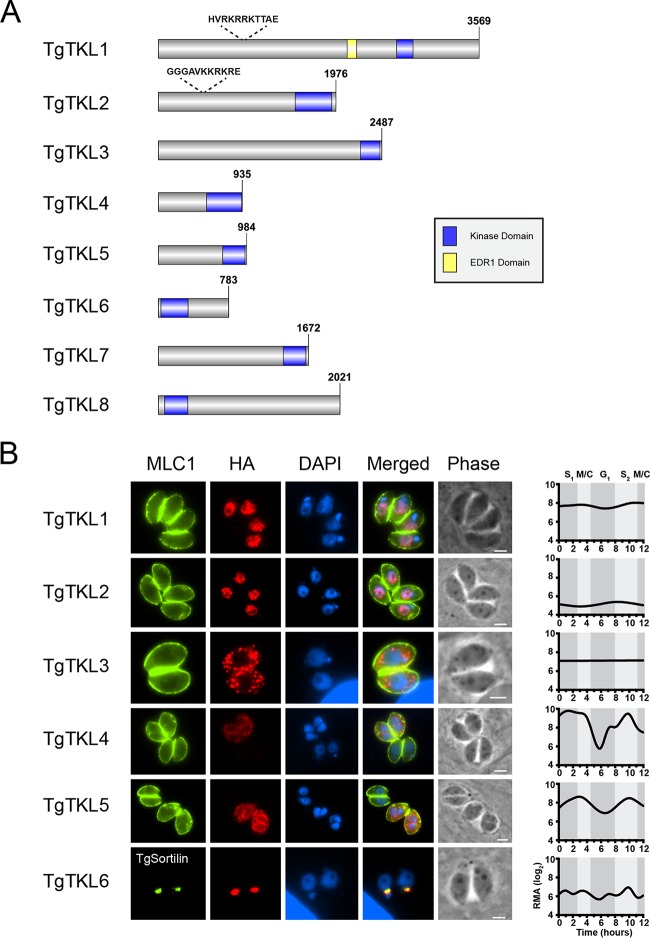
Toxoplasma TKL kinases localize to multiple subcellular regions. (A) Schematic representation of TgTKL proteins, highlighting locations of conserved kinase (blue) and EDR1 (yellow) domains. Putative NLS sequences are shown above TgTKL1 and TgTKL2 data. (B) Immunofluorescence analysis showing localization of six endogenously tagged *Toxoplasma* TKL proteins. TgTKLs were visualized by staining with anti-HA antibody. Myosin light chain (MLC1) was used as a marker for the IMC, and TgSortilin was used as a marker for the Golgi apparatus. TgTKL1 to TgTKL6 were all found to have negative relative fitness scores in a genome-wide CRISPR screen (37), suggesting that they have an important function in *T. gondii* viability. Transcript levels for each gene throughout the cell cycle as reported in reference 38 are shown at the right. Scale bar, 2 μm. RMA, robust multiarray average.

The members of the tyrosine kinase-like (TKL) group of kinases share sequence homology with tyrosine kinases but are catalytically serine-threonine kinases (35). A query of the *Toxoplasma* genome database (http://www.toxodb.org, release 34) identified eight putative TgTKL proteins (36). On the basis of a recent genome-wide loss-of-function screen, six of the eight *Toxoplasma* TKLs appear to be important for tachyzoite growth, while two appear to be dispensable (37). Accordingly, we named the eight putative TKLs in *Toxoplasma* (TgTKL1 to TgTKL8) on the basis of their reported fitness scores, with the strongest contributor to parasite fitness of the TgTKLs being named TgTKL1 (Table 1). All eight TgTKLs contain putative serine-threonine kinase domains, while TgTKL1 also contains an enhanced disease resistance 1 (EDR1) domain (Fig. 1A). Additionally, both TgTKL1 and TgTKL2 contain putative monopartite nuclear localization signal (NLS) sequences, suggesting that these two kinases may localize to the parasite nucleus. Previous studies have shown that, while TgTKL1, TgTKL2, TgTKL3, TgTKL6, TgTKL7, and TgTKL8 are constitutively expressed, TgTKL4 and TgTKL5 are regulated at the transcript level in a cell cycle-dependent manner, with both peaking during late S/early M phase (Fig. 1B) (38). Additionally, four of the eight TgTKLs are differentially expressed between the acute and chronic stages of murine infection; TgTKL8 expression is increased during chronic infection, while the expression of TgTKL1, TgTKL2, and TgTKL5 is decreased during chronic infection (39). Together, these data suggest that TKL kinases may have important functions in multiple stages of the *Toxoplasma* asexual life cycle.

**TABLE 1:** Accession numbers and fitness scores for putative *Toxoplasma* TKL kinases

Gene ID	Fitness score^a^	Kinase name
TGGT1_301270	−5.18	TgTKL1
TGGT1_234970	−4.96	TgTKL2
TGGT1_253860	−2.49	TgTKL3
TGGT1_237210	−1.26	TgTKL4
TGGT1_209050	−0.62	TgTKL5
TGGT1_236240	−0.57	TgTKL6
TGGT1_239130	0.07	TgTKL7
TGGT1_225770	0.30	TgTKL8

a Data represent mean CRISPR phenotype scores reported by Sidik et al. (37).

To explore these potential functions, we first sought to determine where the six TgTKLs that have been suggested to be important for tachyzoite growth localize within the parasite. Accordingly, we introduced a 3× hemagglutinin (HA) epitope tag at the 3′ end of the endogenous gene loci in the RHΔ*Ku80* parasite strain (40). Western blot analysis using an anti-HA antibody revealed a single band of the expected size for each protein in the endogenously HA-tagged clones but not in parental (RHΔ*Ku80*) parasites (see Fig. S1 in the supplemental material). Immunofluorescence analysis (IFA) using anti-HA antibody revealed that TgTKL1 to TgTKL6 localize to different subcellular compartments in *Toxoplasma* tachyzoites, including the nucleus (TgTKL1 and TgTKL2), the cytosol (TgTKL3 and TgTKL4), the inner membrane complex (IMC) (TgTKL5), and the Golgi apparatus (TgTKL6) (Fig. 1B). Importantly, our IFA confirmed that both TgTKL4 and TgTKL5 were expressed only in parasites undergoing cell division, in agreement with their reported regulation at the transcript level (Fig. 1B).

10.1128/mBio.00301-18.1 FIG S1  Confirmation of endogenously tagged TgTKL proteins. Western blot analysis of RHΔKu80 and TgTKL1-HA, TgTKL2-HA, TgTKL3-HA, TgTKL4-HA, TgTKL5-HA, and TgTKL6-HA parasite clone lysates was performed using anti-HA antibody. SAG1 was used as the loading control. Download FIG S1, PDF file, 0.4 MB. Copyright © 2018 Varberg et al. 2018 Varberg et al. This content is distributed under the terms of the Creative Commons Attribution 4.0 International license.

### TgTKL1 is a novel, plant-like nuclear kinase required for efficient parasite growth *in vitro.*

**FIG 2:**
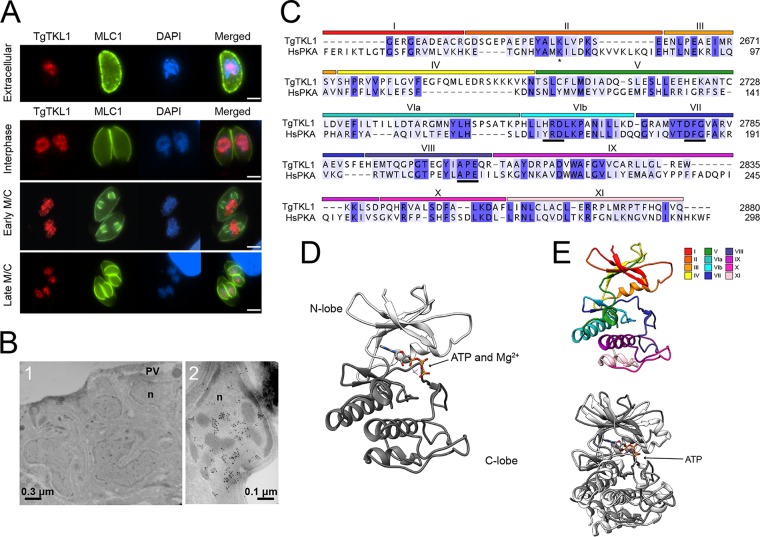
TgTKL1 is a nuclear kinase. (A) The localization of TgTKL1 in extracellular and intracellular parasites was visualized using IFA and staining with an anti-HA antibody. Myosin light chain 1 (MLC1), which localizes to the pellicle, is used as a marker in extracellular parasites. IMC3, which localizes to the inner membrane complex (IMC), is used as a marker in intracellular parasites to visualize progression through different cell cycle stages. Scale bar, 2 μm. (B) Immunoelectron microscopy of intracellular TgTKL1-HA using anti-HA antibody conjugated to gold particles showed that the protein localizes to the nuclei (n) of the parasites in the parasitophorous vacuole (PV) (panel 1), and higher magnification showed that the protein is largely localized to the euchromatin region (panel 2). (C) The amino acid sequence of the TgTKL1 kinase domain (residues 2620 to 2885) was aligned with the kinase domain of *Homo sapiens* protein kinase A (HsPKA) (residues 44 to 298), using ClustalOmega in the JalView v.2 software package. Regions of sequence conservation based on percent identity are illustrated by blue shading, and the locations of structural domains (I to XI) based on the sequence of PKA are shown at the top. (D) Predicted structure of TgTKL1 kinase domain, highlighting the ATP binding site and the N-lobe and C-lobe. (E) Visual representation of the conserved structural domains in the predicted TgTKL1 kinase domain (top) and overlay of predicted TgTKL1 kinase domain (dark gray) with the PKA (light gray) crystal structure (bottom) (PDB ID 4WB5) (root mean square deviation [RMSD] = 1.055 Å).

We next focused our efforts on the functional characterization of TgTKL1, as it has been suggested to be the strongest contributor to parasite fitness. TgTKL1 is 3,569 amino acids in length and contains a C-terminal kinase domain (residues 2631 to 2883) and an EDR1 domain (residues 2101 to 2213) (Fig. 1A). In plants, EDR1 domain-containing kinases, such as the *Arabidopsis* kinases EDR1 and CTR1, have been shown to play a role in stress response pathways, including ethylene-mediated signaling (41–43). Importantly, EDR1 domain-containing kinases are uniquely found in plants and protozoans, making them attractive candidates for drug development. Our initial IFA results showed that TgTKL1 localizes to the nucleus in intracellular parasites (Fig. 1B). Further analysis revealed that TgTKL1 is retained in the nucleus throughout the parasite cell cycle, as well as in extracellular parasites (Fig. 2A). To further confirm our IFA findings, we performed immunoelectron microscopy with an anti-HA antibody conjugated to gold particles. The results showed that the signal was localized to the parasite nucleoplasm in the parasitophorous vacuole (PV) (Fig. 2B1; see also Fig. S2) and that the protein predominantly associates with the euchromatin region within the nucleus (Fig. 2B, panel 2).

10.1128/mBio.00301-18.2 FIG S2  TgTKL1 localizes to the nucleus. The image is an enlarged version of the immuno-electron microscopy (EM) image shown in panel 1 in Fig. 2B (top panel), and a representative image of an additional region is shown at lower magnification in the bottom panel. TgTKL1-HA was visualized by staining with anti-HA antibody followed by labeling with a gold-conjugated secondary antibody. Download FIG S2, PDF file, 2.3 MB. Copyright © 2018 Varberg et al. 2018 Varberg et al. This content is distributed under the terms of the Creative Commons Attribution 4.0 International license.

Alignment of the TgTKL1 kinase domain sequence with the canonical cyclic AMP (cAMP)-dependent protein kinase (PKA) showed that all of the residues typically required for catalytic activity were present in TgTKL1 (Fig. 2C). These include the DFG and HRD motifs present in the activation and catalytic loops, respectively, as well as the APE motif and the invariant lysine residue corresponding to PKA Lys^73^ (asterisk, Fig. 2C) (44). In many kinases, catalytic activity is increased by autophosphorylation of residues typically found within 20 amino acids of the APE motif (44). Interestingly, two of the reported sites of phosphorylation on TgTKL1 (residues S2789 and T2795) fall within 16 amino acids of the APE motif, suggesting that these modifications may contribute to regulation of TgTKL1 catalytic activity. Lastly, kinase substrate specificity is conferred by conserved sequence motifs immediately downstream of the HRD motif and immediately upstream of the APE motif (44). Examination of these regions in TgTKL1 suggests that TgTKL1 is a member of the serine/threonine kinase family.

Structural modeling of the TgTKL1 kinase domain using the I-TASSER server (45) predicted that this domain would share features typical of ePKs, including an N-terminal lobe predominantly composed of beta-sheets and a C-terminal lobe that is mostly alpha-helical in nature (Fig. 2D). The conservation of amino acid sequence and predicted tertiary structure extends throughout all 12 canonical kinase subdomains (46) and shows a high degree of similarity with the resolved PKA kinase domain structure (46, 47) (Fig. 2C and E).

**FIG 3:**
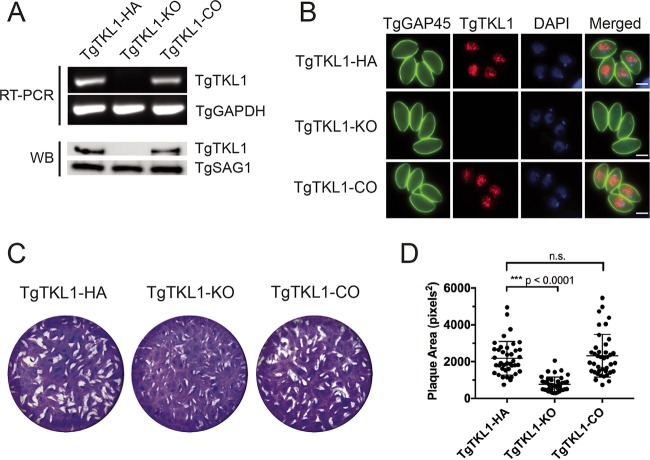
Generation of TgTKL1 knockout and complemented strains. (A and B) Semiquantitative RT-PCR and Western blot (WB) analysis (A) and immunofluorescence analysis (B) of wild-type (TgTKL1-HA), knockout (TgTKL1-KO), and complemented (TgTKL1-CO) parasites. For RT-PCR, primers that amplify parasite GAPDH (glyceraldehyde-3-phosphate dehydrogenase) were used as controls. For Western blotting, SAG1 was used as the loading control. In IFAs, TgTKL1 (red) was visualized by staining with anti-HA antibody, while TgGAP45 (green) was used as a marker for the IMC. Scale bar, 2 μm. (C) Plaque assay examining the growth of wild-type (TgTKL1-HA), knockout (TgTKL1-KO), and complemented (TgTKL1-CO) parasites in HFF cells. Plaques are visible as clear zones on the background of a crystal violet-stained HFF monolayer. (D) Quantification of plaque area sizes of TgTKL1-HA, TgTKL1-KO, and TgTKL1-CO strains. ***, *P* < 0.0001; n.s., not significant.

We next sought to determine the role of TgTKL1 in the lytic cycle biology of *Toxoplasma*. With this goal in mind, we generated TgTKL1 knockout (TgTKL1-KO) and TgTKL1 complemented (TgTKL1-CO) strains as described in Materials and Methods. All strains were validated for the presence or absence of TgTKL1 using reverse transcription-PCR (RT-PCR), Western blotting, and immunofluorescence analysis (Fig. 3A and B). With these reagents in hand, we next examined the effect of the loss of TgTKL1 on parasite growth *in vitro* by monitoring the formation of plaques on the confluent human foreskin fibroblast (HFF) monolayer that results from multiple rounds of parasite invasion, replication, and egress. We observed a significant reduction in the size of plaques formed by TgTKL1-KO parasites compared to TgTKL1-HA parasites (Fig. 3C and D). Importantly, this reduction in plaque size was restored in the TgTKL1-CO parasite strain (Fig. 3C and D). These results indicate that the novel nuclear kinase TgTKL1 plays an important role in *Toxoplasma* tachyzoite growth *in vitro*.

### Loss of TgTKL1 impairs microneme secretion, processing, and host cell attachment.

**FIG 4:**
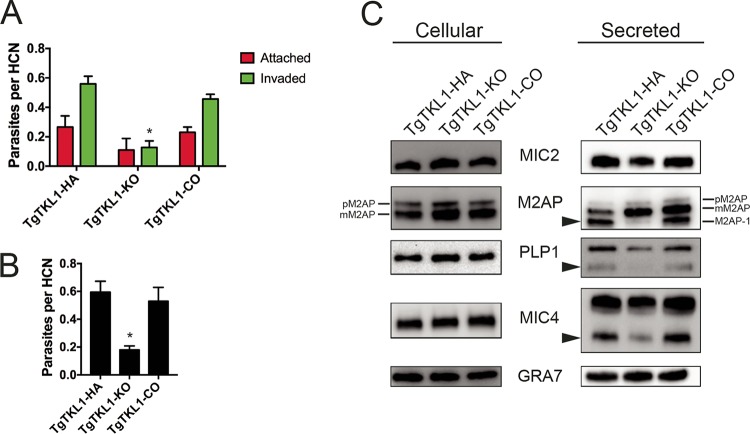
TgTKL1 is required for efficient microneme processing and host cell attachment. (A) Red-green invasion assay of *Toxoplasma* tachyzoites after 30 min of incubation with HFF cells. Data represent averages of results from three independent experiments each performed with technical triplicates ± standard errors of the means (SEM). *, *P* < 0.05. HCN, host cell nucleus. (B) Attachment of mycalolide B-treated tachyzoites to HFF cells. Parasites were treated for 10 min with mycalolide B, washed, and then added onto HFF cells. At 30 min postincubation at 37°C, slides were washed, fixed, and stained and the numbers of attached parasites were quantified. Data represent averages of results from three independent experiments each performed with technical triplicates ± SEM. *, *P* < 0.05. (C) Parasite lysate (cellular fraction) and ESA fractions from wild-type (TgTKL1-HA), knockout (TgTKL1-KO), and complemented (TgTKL1-CO) strains. ESA fractions were collected from culture supernatants after parasites were treated for 2 min at 37°C with 1% ethanol to stimulate microneme secretion. GRA1 was immunoblotted as the loading control for each strain. pM2AP, Pro M2AP; mM2AP, mature M2AP; M2AP-1, M2AP processed 1.

Impairment of plaque formation can be caused by defects in one or more steps of the parasite lytic cycle. Therefore, we next sought to determine which aspect of the lytic cycle was impaired in TgTKL1-KO parasites. We first assessed parasite replication using standard doubling assays (48) and found no significant differences in the replication rates seen with TgTKL1-HA and TgTKL1-KO strains (data not shown). Likewise, the loss of TgTKL1 did not alter the ability for parasites to egress from the host cell in response to treatment with the ionophore A23187 (data not shown). However, when we performed red-green invasion assays (49) to assess the host cell attachment and invasion processes, we observed a significant (~60%) decrease in the number of invaded parasites in TgTKL1-KO parasites compared to wild-type parasites (Fig. 4A). Importantly, the invasion competence was restored by TgTKL1 complementation (Fig. 4A). In this assay, parasites that have defects in the active invasion process show a decrease in the number of invading (intracellular) parasites along with a concomitant increase in the number of attached (extracellular) parasites. In contrast, specific defects in the host cell attachment process result in a decrease in the numbers of both attached and invaded parasites. As the TgTKL1-KO parasites did not show an increase in the number of attached parasites (Fig. 4A), we hypothesized that the loss of TgTKL1 specifically impairs the process of host cell attachment.

To test this hypothesis, we performed host cell attachment assays using parasites treated with mycalolide B, an irreversible inhibitor of actin polymerization (50). Toxoplasma tachyzoites treated with this drug are able to secrete micronemal proteins and attach to the host cell but cannot penetrate and complete the invasion process, as actin polymerization is critical for parasite motility (51). These assays revealed that, in comparison with TgTKL1-HA parasites, TgTKL1-KO parasites showed a significant reduction (~66%) in attachment and that the level of attachment was restored to wild-type levels upon TgTKL1 complementation (Fig. 4B). These results showed that the invasion phenotype observed in TgTKL1-KO parasites is specifically due to impairment of the host cell attachment process.

Previous studies have established that the attachment step of *Toxoplasma* invasion is dependent on the effective secretion of micronemal proteins by the parasite (52). As TgTKL1-KO parasites exhibited a defect in attachment, we next sought to determine whether this defect was due to alterations in the expression or secretion of micronemal proteins. We first assessed the expression of micronemal proteins MIC2, M2AP, PLP1, and MIC4 by Western blotting (53–57). As a control, we also probed the membranes using an antibody against the dense granule GRA1 protein that is constitutively secreted by the parasite (58). Interestingly, these assays revealed that all of the micronemal proteins that we examined were expressed at similar levels across TgTKL1 wild-type, knockout, and complemented strains (Fig. 4C).

We next treated all three parasite strains with ethanol to stimulate microneme secretion (59), and the secreted fraction was subjected to Western blotting. We observed that the secretion of micronemal proteins MIC2, M2AP, PLP1, and MIC4, despite their being expressed at equivalent levels, was decreased in TgTKL1-KO parasites compared to TgTKL1-HA and TgTKL1-CO strains (Fig. 4C; see also Fig. S3). Additionally, TgTKL1-KO parasites displayed significant defects in the postexocytosis surface processing of M2AP, MIC4, and PLP1 (Fig. 4C, arrowheads; see also Fig. S3). These processing events occur after the micronemal proteins are trafficked to and released onto the parasite surface, and their processing is required for efficient host cell attachment (60, 61). Importantly, trafficking of these three proteins appears to be unaffected by loss of TgTKL1 (Fig. S4). Together, these results suggest that the *in vitro* growth defects observed in TgTKL1-KO parasites are due to impairment of the host cell attachment process caused by defects in the secretion and processing of micronemal proteins.

10.1128/mBio.00301-18.3 FIG S3  TgTKL1 is involved in microneme secretion and postexocytosis processing. (A) Quantification of relative total amounts of micronemal proteins (MIC2, M2AP, PLP1, and MIC4) secreted in TgTKL1-HA, TgTKL1-KO, and TgTKL1-CO strains following treatment with ethanol. The constitutively secreted dense granule GRA1 protein was used as a control. The ratio of total signals from all the bands of each microneme protein to the GRA1 signal was determined for each of the strains, and the ratio obtained with the TgTKL1-HA clone was set at 100%. The quantification of signal was performed using a FluorChem E system (ProteinSimple). Data were compiled from results from three independent experiments, and error bars represent SEM. *, *P* < 0.05. (B) Quantification of relative levels of microneme processing of M2AP, PLP1, and MIC4 in ethanol-induced TgTKL1-HA, TgTKL1-KO, and TgTKL1-CO strains. The constitutively secreted GRA1 was used as a control. The ratio of the smaller processed band signal of each of the microneme proteins to the GRA1 signal was determined, and the ratio obtained for TgTKL1-HA was set at 100%. The quantification of signal was performed using a FluorChem E system (ProteinSimple). Data were compiled from results from three independent experiments, and error bars represent SEM. *, *P* < 0.05. Download FIG S3, PDF file, 0.3 MB. Copyright © 2018 Varberg et al. 2018 Varberg et al. This content is distributed under the terms of the Creative Commons Attribution 4.0 International license.

10.1128/mBio.00301-18.4 FIG S4  Microneme trafficking is not altered in TgTKL1-KO parasites. IFA of M2AP (A), MIC4 (B), and PLP1 (C) in TgTKL1-HA, TgTKL1-KO, and TgTKL1-CO parasites was performed. All three proteins properly localized to the apical region of the parasite, suggesting that intracellular trafficking is not TgTKL1 dependent. The inner membrane complex was visualized with antibodies against IMC1 (red), and nuclei were visualized by staining with DAPI (blue). Scale bar, 2 μm. Download FIG S4, PDF file, 1.9 MB. Copyright © 2018 Varberg et al. 2018 Varberg et al. This content is distributed under the terms of the Creative Commons Attribution 4.0 International license.

### TgTKL1 disruption results in global changes in gene expression.

**FIG 5:**
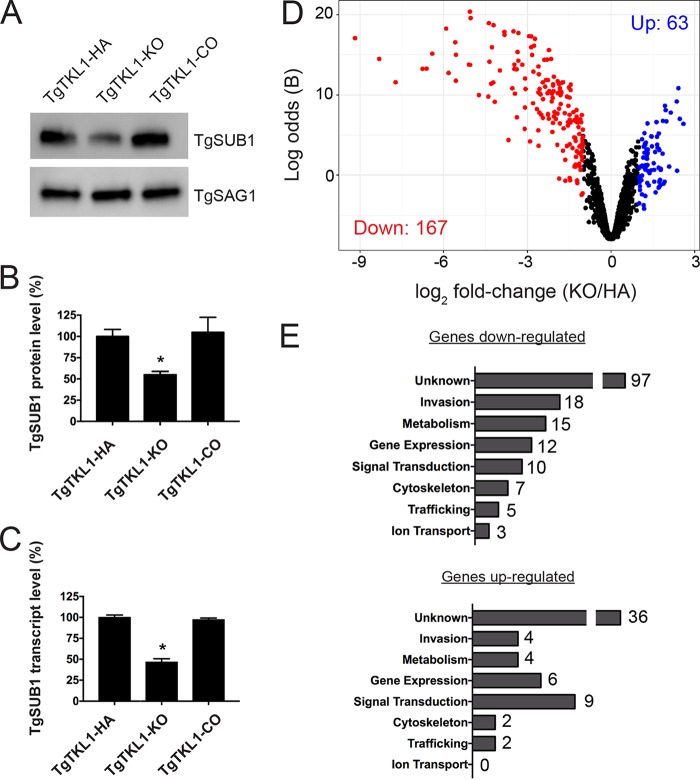
Loss of TgTKL1 alters the expression of multiple invasion-related factors. (A) Representative Western blot image of TgTKL1-HA, TgTKL1-KO, and TgTKL1-CO parasite lysates with anti-TgSUB1 antibody. SAG1 is used as the loading control. (B) Quantification of TgSUB1 protein levels in TgTKL1-HA, TgTKL1-KO, and TgTKL1-CO strains. The quantification of protein signal was determined using a FluorChem E (ProteinSimple) system. Data represent results from three independent experiments, and error bars represent SEM. *, *P* < 0.05. (C) Quantification of TgSUB1 transcript levels in TgTKL1-HA, TgTKL1-KO, and TgTKL1-CO strains by qRT-PCR. Data represent averages of results from three independent experiments each performed with technical triplicates ± SEM. *, *P* < 0.05. (D) Volcano plot of statistical significance (log odds ratio, B) versus fold change (log_2_), highlighting genes identified as differentially expressed (|log_2_ fold change| ≥ 1, FDR ≤ 1%) in TgTKL1-KO parasites compared to TgTKL1-HA parasites. Downregulated genes (*n* = 167) are highlighted in red, and upregulated genes (*n* = 63) are highlighted in blue. (E) Functional classification of the 167 genes downregulated and 63 genes upregulated in TgTKL1-KO parasites. Classifications were manually assigned according to known gene functions or putative functions based on conserved domains.

Many micronemal proteins are cleaved at the parasite surface after secretion by the protease TgSUB1, and loss of TgSUB1 significantly impairs host cell attachment (62). Since we observed defects in microneme processing and in attachment of the parasite to the host cells in TgTKL1-KO parasites, we next sought to determine whether these defects could be attributed to alterations in the expression of TgSUB1. Immunoblotting for TgSUB1 revealed that the TgSUB1 protein levels in TgTKL1-KO parasites were significantly reduced compared to wild-type parasites and that the levels were restored by TgTKL1 complementation (Fig. 5A and B). Interestingly, quantitative reverse transcription-PCR (qRT-PCR) analysis showed that TgSUB1 transcript levels were also reduced ~50% in TgTKL1-KO parasites, suggesting that TgTKL1 may regulate TgSUB1 at the transcription level (Fig. 5C). This was a particularly exciting finding as it provides a plausible mechanistic explanation for how TgTKL1 could affect host cell attachment and invasion while residing in the nucleus.

Our observation of a potential role for TgTKL1 in transcriptional regulation led us to examine whether TgTKL1 had a broader function in regulating gene expression. To test this, we purified RNA from TgTKL1-HA and TgTKL1-KO parasites and performed transcriptomics analysis to assess the effects of TgTKL1 knockout on gene expression globally. These studies identified 230 genes that were significantly differentially expressed (|log_2_ fold change [log_2_FC]| ≥ 1, false-discovery rate [FDR] ≤ 1%) in the TgTKL1-KO mutants compared to the TgTKL1-HA parasites (Fig. 5D; see also Data Set S1 in the supplemental material). Of these 230 differentially expressed genes, 167 genes were downregulated in TgTKL1-KO parasites whereas 63 genes were upregulated. We next manually assigned functional classifications for the 230 differentially expressed genes on the basis of their known or putative functions (Fig. 5E; see also Data Set S1). The results revealed that the downregulated gene set contained numerous invasion-related genes, including those encoding surface proteins, micronemal proteins, and rhoptry proteins. In agreement with our previous studies, the list of 167 downregulated genes included TgSUB1 (log_2_FC = −1.2). To further validate our data set, the expression levels of four genes that were identified as downregulated in TgTKL1-KO parasites, three genes identified as upregulated, and an unaffected gene were assessed by qRT-PCR. Importantly, in addition to validation of the changes in expression levels between TgTKL1-HA and TgTKL1-KO strains, all genes were found to be expressed at wild-type levels in TgTKL1-CO parasites (Fig. S5). Together, these findings suggest that TgTKL1 has a broader role in coordinating the expression of numerous genes in *Toxoplasma* tachyzoites. Further, our transcriptomics analyses identified numerous attachment-and-invasion-related factors that are significantly downregulated in TgTKL1-KO parasites and that likely contribute to the observed attachment and invasion defects in these parasites.

10.1128/mBio.00301-18.5 FIG S5  Validation of RNA sequencing results by qRT-PCR. Changes in gene expression identified by RNA sequencing results were confirmed by qRT-PCR analysis for four genes that were downregulated in TgTKL1-KO parasites (top panel), three genes that were upregulated in TgTKL1-KO parasites (bottom panel), and one gene whose regulation did not change in TgTKL1-KO parasites (bottom panel) compared to TgTKL1-HA parasites. Data represent results from three independent biological replicates, and error bars represent SEM. *, *P* < 0.05. Download FIG S5, PDF file, 0.6 MB. Copyright © 2018 Varberg et al. 2018 Varberg et al. This content is distributed under the terms of the Creative Commons Attribution 4.0 International license.

10.1128/mBio.00301-18.9 DATA SET S1  Spreadsheet containing transcriptomics analyses and functional classification for genes that are differentially expressed in TgTKL1-KO parasites. Download DATA SET S1, XLSX file, 0.1 MB. Copyright © 2018 Varberg et al. 2018 Varberg et al. This content is distributed under the terms of the Creative Commons Attribution 4.0 International license.

### TgTKL1 is essential for parasite virulence in mice.

**FIG 6:**
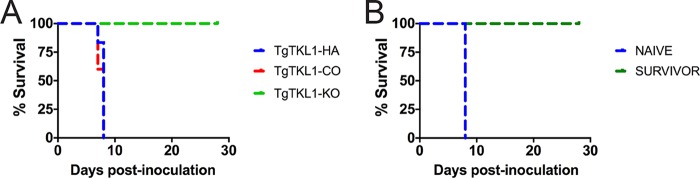
TgTKL1 is required for virulence in mice. (A) Mice were injected intraperitoneally with 500 tachyzoites of wild-type (TgTKL1-HA), knockout (TgTKL1-KO), or complemented (TgTKL1-CO) strains (five mice for each strain). Concomitant plaque assays were performed to corroborate the numbers of viable tachyzoites injected. (B) Naive mice and mice that survived TgTKL1 knockout parasite infection were rechallenged with 500 wild-type tachyzoites with 5 mice in each group.

Previous studies have shown that *Toxoplasma* parasites with deletions of invasion-related genes, including those encoding microneme and rhoptry proteins, exhibited attenuated virulence *in vivo* (22, 24, 28, 30, 49, 62–64). Therefore, we hypothesized that TgTKL1-KO parasites would exhibit a similar decrease in virulence and sought to assess the effects of TgTKL1 knockout using a mouse model of acute toxoplasmosis. Accordingly, five female CBA/J mice were infected with 500 parasites of TgTKL1 wild-type, knockout, or complemented parasites via intraperitoneal injection. Remarkably, while all of the mice injected with either wild-type or complemented parasites died within 10 days of infection, all of the mice injected with TgTKL1 knockout parasites survived (Fig. 6A). Analysis of serum samples from the survivor mice at 3 weeks postinfection confirmed parasite exposure, as all mice were found to be seropositive (data not shown). To determine whether exposure to the TgTKL1 knockout strain provides protective immunity, we challenged five naive mice and the five surviving TgTKL1-KO-exposed mice with 500 wild-type parasites. All of the naive mice succumbed to *Toxoplasma* infection within the first 10 days following injection, while all the TgTKL1-KO-exposed mice survived the rechallenge (Fig. 6B). These results show that TgTKL1 is an essential virulence factor *in vivo* and that, despite the loss of lethality, inoculation with TgTKL1-KO parasites provides protective immunity to future exposures.

## DISCUSSION

Protein kinases have emerged as being among the most highly targeted classes of proteins for drug development due to their roles in regulating numerous aspects of eukaryotic cell biology. Importantly, kinases also regulate multiple essential processes in *Toxoplasma* and other protozoan parasites. Accordingly, they have received a significant amount of interest as potential drug targets for novel antiparasitics. In this study, we sought to characterize the previously unexplored class of TKL kinases in *Toxoplasma*. Endogenous tagging of the six TgTKLs that have recently been shown to be important for tachyzoite fitness *in vitro* showed that members of this group localize to multiple subcellular compartments, including the nucleus, the cytoplasm, the IMC, and the Golgi apparatus. As all of the TgTKLs are predicted to be catalytically active (26), we believe that it is likely that these kinases regulate numerous aspects of parasite biology through their enzymatic activity. Further, potential functions can be predicted for the few TgTKLs that have putative orthologues in higher eukaryotes. For example, TgTKL2 is a putative orthologue of Tousled-like kinase 2 (Tlk2), a nucleus-localized kinase with peak activity in S phase that promotes DNA replication by phosphorylation of the chromatin association factor AsfI (65–67). Tousled-like kinases (Tlks) are also associated with the DNA damage response and are phosphorylated by ATM and Chk1 kinases in response to DNA double-strand breaks (68). Although Tlks have been studied only in multicellular organisms, it is possible that TgTKL2 has similar functions in DNA replication and cell cycle progression in *Toxoplasma*. Additionally, the function of TgTKL6 might be similar to that of its putative mammalian orthologue, cyclin G-associated kinase (GAK). Like TgTKL6, GAK localizes to the Golgi apparatus, where it assists in the disassembly of clathrin-coated vesicles (69). Future studies characterizing the function of additional TgTKLs, as well as the identification of their substrates, are required to determine the exact function of these kinases in the parasite. Such studies will likely shed light on the role of TgTKLs in regulating essential cellular processes in *Toxoplasma* while also exploring the conserved or divergent functions of these proteins throughout eukaryotic evolution.

Perhaps even more intriguing are the TgTKLs lacking orthologues in higher eukaryotes, as these kinases likely have functions in parasite-specific biological processes and thus may be ideal targets for drug development. This group includes TgTKL4 and TgTKL5, which we have shown are uniquely expressed in parasites undergoing cell division (Fig. 1). As progression through the cell cycle is known to be regulated by phosphorylation in many eukaryotic systems, it is plausible that these two TKLs play similar regulatory roles in *Toxoplasma*. The localization of TgTKL5 to the IMC specifically during cell division is especially exciting and suggests that this kinase may have a role in the formation of the daughter parasite pellicle during endodyogeny. Also of interest are the four TgTKLs that are differentially expressed between the acute and chronic stages of disease, as these kinases may have important functions in the parasite stage differentiation required for establishing and maintaining chronic infections. For example, TgTKL8 expression increases 2-fold during chronic infection compared to the acute stage (39). Accordingly, it is possible that the function of this kinase is specifically required in bradyzoites and is dispensable in tachyzoites, thus explaining its positive fitness score in the genome-wide clustered regularly interspaced short palindromic repeat (CRISPR) screen performed on tachyzoites. Future studies are needed to explore the role of these kinases throughout intracellular development and with respect to stage differentiation.

Like TgTKL4 and TgTKL5, nucleus-localized TgTKL1 lacks a mammalian homologue, making it an attractive drug target. Further, TgTKL1 contains both an EDR1 domain and a protein kinase domain, a domain architecture uniquely found in plants and protozoans. While little is known regarding the biological function of the EDR1 domain, characterization of the EDR1 domain-containing kinases EDR1 and CTR1 in *Arabidopsis* showed that these serine/threonine kinases have important roles in hormone-responsive signal transduction pathways activated by various stressors (70–72). Since we observed that TgTKL1 localized exclusively at the nucleus (Fig. 2), we hypothesized that this plant-like kinase may have a signaling function within the nucleus and sought to begin our characterization of the TgTKLs by functionally characterizing TgTKL1.

In agreement with its reported importance for tachyzoite fitness (37), we observed that TgTKL1 knockout parasites displayed a marked level of growth impairment *in vitro* (Fig. 3). Previous studies had also shown that the mRNA levels of TgTKL1 are slightly upregulated in extracellular parasites, suggesting that this gene might be involved in parasite invasion (50). Indeed, our studies revealed that the growth impairment in TgTKL1 knockout parasites was caused by defects in host cell attachment and invasion and that parasites lacking TgTKL1 have defects in microneme secretion and processing (Fig. 4). Initially, it was difficult to reconcile the exclusive localization of TgTKL1 within the nucleus with its apparent requirement for efficient microneme secretion and processing. However, our finding that TgSUB1 expression is dependent on TgTKL1 provides a probable explanation for the observed phenotype, whereby TgTKL1 resides in the nucleus and directly phosphorylates nuclear substrates to alter gene expression. This explanation is further supported by our observation that the expression levels of over 200 genes are significantly altered in parasites lacking TgTKL1 (Fig. 5; see also Data Set S1). While TgTKL1 substrates remain unknown, it is feasible that TgTKL1 might be phosphorylating factors involved in regulation of gene expression, such as chromatin-modifying enzymes and/or the plant-like AP2 transcription factors. Interestingly, previous studies have shown that genes encoding micronemal proteins share common *cis*-acting elements in the promoter region that play an essential role in their expression (73). Hence, it is plausible that the one or more transcription factors phosphorylated by TgTKL1 could recognize motifs upstream of the differentially expressed genes in TgTKL1 knockout parasites. Future studies employing a combination of bioinformatics and experimental approaches to analyze the promoter regions of TgTKL1-regulated genes may allow identification of conserved *cis*-acting motifs, as well of the factors that bind to these regions.

Our *in vitro* studies showed that TgTKL1 knockout parasites exhibit a severe defect in host cell attachment due to impaired microneme secretion and processing. Although we anticipated that this might result in attenuation of virulence in mice, we were surprised to discover that TgTKL1 knockout parasites were completely avirulent (Fig. 6). The observed decrease in TgSUB1 expression provides a plausible explanation for the observed defects in micronemal processing. However, we do not believe that the complete loss of virulence in TgTKL1 knockout parasites can be solely attributed to the impaired microneme processing caused by reduced levels of TgSUB1, as complete loss of TgSUB1 resulted in only partial attenuation of virulence in mice (62). Our transcriptomics analysis identified numerous attachment-and-invasion-related factors that are significantly downregulated in TgTKL1 knockout parasites (Fig. 5; see also Data Set S1). In addition, even though many of the micronemal proteins are expressed at normal levels in TgTKL1 knockout parasites, their secretion is decreased in TgTKL1 knockout parasites, thus leading to further impairment of the attachment process. Thus, it is possible that the described severe *in vivo* phenotypes are the result of cumulative effects caused by global dysregulation of proteins involved in host cell attachment and invasion. Alternatively, it is possible that one of the uncharacterized genes downregulated in TgTKL1 knockouts represents a novel, essential virulence factor. It is noteworthy that none of the established virulence factors (2, 74, 75) were found to be downregulated in the TgTKL1 knockout parasites, further suggesting that the loss of virulence either is due to the decreased expression of a novel virulence factor or is a multifactorial effect of global alterations in gene expression. However, although we did not observe alterations in transcript levels for known virulence factors, additional studies are required to determine whether TgTKL1 is required for their functionality. Nonetheless, our functional characterization of TgTKL1 shows that this novel, plant-like kinase regulates the expression of numerous genes, including those encoding multiple attachment-and-invasion-related factors, and is an essential virulence factor in *Toxoplasma*.

## MATERIALS AND METHODS

### Parasite cultures.

*Toxoplasma gondii* tachyzoites were maintained by passage through human foreskin fibroblasts (HFF) in a humidified incubator at 37°C with 5% CO_2_. Normal growth medium consisted of Dulbecco’s modified Eagle’s medium (DMEM) supplemented with 10% fetal bovine serum, 2 mM l-glutamine, and 50 μg/ml of penicillin-streptomycin. Purification of parasites was performed as previously described (50).

### Identification of TgTKL domains and NLS sequences.

To identify conserved domains, TgTKL protein sequences were searched against the Pfam database (v. 31.0; http://pfam.xfam.org/) with an E value cutoff of 1 × 10^−5^ (76, 77). Putative NLS sequences were identified using the cNLS mapper tool (78) and were considered to represent high confidence when the score was ≥10 (79).

### Endogenous tagging of TgTKL genes.

TgTKL1 to TgTKL6 were endogenously tagged at their C terminus using the methods described in reference 40. Briefly, genomic DNA was purified from RHΔ*Ku80* parasites and used as a template to amplify fragments upstream of the stop codon of TgTKL1 to TgTKL6. The primers used for amplification are listed in Table S1 in the supplemental material. The PCR products were then cloned into the PacI site of plasmid pLIC.HA3.DHFR (40) using an In-Fusion HD cloning kit (Clonetech). The resulting plasmids were linearized and electroporated into RHΔ*Ku80* parasites (80, 81). The transfected parasites were cultured in the presence of pyrimethamine (1 μM) (82) to select for drug-resistant parasites, and single clones were then isolated by limiting dilution.

10.1128/mBio.00301-18.6 TABLE S1  Primers used for tagging TKL genes in *Toxoplasma gondii*. Download TABLE S1, DOCX file, 0.1 MB. Copyright © 2018 Varberg et al. 2018 Varberg et al. This content is distributed under the terms of the Creative Commons Attribution 4.0 International license.

### Immunofluorescence microscopy.

Immunofluorescence staining of intracellular parasites was performed according to a previously described procedure (83). The primary antibodies used were mouse anti-HA antibody (Cell Signaling Technology, Inc.) (6E2; 1:1,000), mouse anti-SAG1 antibody (Genway) (1:1,000), rabbit anti-GAP45 antibody (1:1,000) (84), and rabbit anti-IMC3 antibody (1:2,000) (85). The secondary antibodies used included Alexa Fluor 594-conjugated or Alexa Fluor 488-conjugated goat anti-rabbit antibody or goat anti-mouse antibody (Molecular Probes) (1:1,000). Slides were viewed using a Nikon Eclipse E100080i microscope, and digital images were captured with a Hamamatsu C4742-95 charge-coupled-device camera using NIS-elements software.

### Immunoelectron microscopy.

For immunoelectron microscopy of TgTLK1, HFF were infected with *Toxoplasma* expressing TgTLK1-HA for 24 h and fixed in 4% paraformaldehyde (Electron Microscopy Sciences, Hatfield, PA)–0.25 M HEPES (pH 7.4) for 1 h at room temperature and then in 8% paraformaldehyde in the same buffer overnight at 4°C. They were infiltrated, frozen, and divided into sections as previously described (86). The sections were immunolabeled with antibodies against HA (1:10) diluted in phosphate-buffered saline (PBS)–1% fish skin gelatin and then with secondary IgG antibodies coupled to protein A-gold particles (size, 15 nm).

### Structural modeling.

The I-TASSER server (45) was used to generate a predicted structure of the TgTKL1 kinase domain, using the amino acid sequence of TgTKL1 (residues 2620 to 2885) as the input. Modeling was performed using default settings and without template specification. The I-TASSER output was visualized using the UCSF Chimera software package (87). The TgTKL1 kinase domain was superimposed on the resolved structure of the PKA alpha subunit (PDB identifier [ID] 4WB5) (47) using the Chimera “MatchMaker” tool (88). Following the superpositioning, the structure-based multiple-sequence alignment was computed using the Chimera “Match -> Align” tool (88). This structure-based sequence alignment was used to map the canonical kinase structural domains (I to XI) to the TgTKL1 structure on the basis of the structural domain sequences reported for PKA (46).

### Generation of TgTKL1 knockout and complemented strains.

A TgTKL1 knockout plasmid construct was made by inserting approximately 1.5-kb regions of sequence homology corresponding to regions 5′ and 3′ of the TgTKL1 coding sequence into the pminiHXGPRT plasmid (89) using the primers listed in Table S2. Briefly, the TgTKL1 5′ flank was amplified and directionally cloned into KpnI/HindIII-digested pminiHXGPRT to generate TgTKL1.5′-HXGPRT. Similarly, the 3′ flank of TgTKL1 was amplified and directionally cloned into BamHI/NotI-digested TgTKL1.5′-HXGPRT to generate the final TgTKL1 knockout construct. Prior to transfection, the DNA fragment containing the HXGPRT expression cassette flanked by the TgTKL1 5′ and 3′ homology regions was excised by digestion with KpnI and NotI, subjected to gel purification, and electroporated into TgTKL1-HA parasites. The parasites were then cultured in the presence of 50 μg/ml mycophenolic acid (MPA) and 50 μg/ml xanthine to select drug-resistant parasites and cloned by limiting dilution (90). The clones were screened by IFA for loss of TgTKL1-HA, and the TgTKL1 knockout clones were further verified by RT-PCR and Western blot analysis.

10.1128/mBio.00301-18.7 TABLE S2  Primers used for generating TKL1 knockout strain. Download TABLE S2, DOCX file, 0.04 MB. Copyright © 2018 Varberg et al. 2018 Varberg et al. This content is distributed under the terms of the Creative Commons Attribution 4.0 International license.

To obtain a TgTKL1 complemented parasite cell line, we used the ToxoDB genomic database to identify cosmid clone PSBLW20 from the pSC/Ble library that contained the TgTKL1 locus. This cosmid also contains the *Ble* gene as a selectable marker that provides resistance to phleomycin (91). TgTKL1 knockout parasites were transfected with the PSBLW20 cosmid and added to an HFF monolayer and were allowed to grow without any drug selection until the monolayer was lysed. Freshly egressed parasites were then washed with Hanks’s balanced salt solution containing 10 mM HEPES and 0.1 mM EGTA (HHE), and extracellular parasites were treated with 50 μg/ml phleomycin–DMEM for 4 h at 37°C with 5% CO_2_. The parasites were then added to an HFF monolayer and cultured in the presence of 5 μg/ml phleomycin to select for drug-resistant parasites, which were then cloned by limiting dilution. Recovered clones were analyzed for TgTKL1 expression by RT-PCR using primers listed in Table S3. To further validate the TgTKL1 complementation, a clone that showed expression of TgTKL1 by RT-PCR was transfected with the TgTKL1 endogenous tagging construct (as described above). These parasites were then grown in the presence of pyrimethamine to generate HA-tagged TgTKL1 complemented clones that were further verified by IFA and Western blotting.

10.1128/mBio.00301-18.8 TABLE S3  Primers used for qRT-PCR analysis. Download TABLE S3, DOCX file, 0.1 MB. Copyright © 2018 Varberg et al. 2018 Varberg et al. This content is distributed under the terms of the Creative Commons Attribution 4.0 International license.

### Plaque assays.

Intracellular parasites were harvested, syringe filtered, and added onto a confluent monolayer of HFF cells in a 12-well plate (500 tachyzoites per well). The plates were then incubated at 37°C for 6 days without any movement. The plates were then washed with PBS, methanol fixed, and stained with 2% crystal violet to visualize regions of host cell disruption.

### Invasion assays.

Invasion assays were performed in eight-well chamber slides as described previously (50) with the following modifications. Purified tachyzoites were added onto HFF monolayers (2 × 10^6^ parasites/well) and incubated at 37°C for 30 min. Slides were then washed three times to remove noninvaded parasites, fixed, blocked, and stained with mouse anti-SAG1 without permeabilization. After 1 h, the slides were washed, permeabilized with 0.01% Triton X-100, and stained with rabbit anti-GAP45 antibody. The slides were further washed and stained with secondary antibodies, namely, Alexa Fluor 594-conjugated goat anti-mouse antibody (Molecular Probes) and Alexa Fluor 488-conjugated goat anti-rabbit antibody (Molecular Probes). After 1 h, slides were washed and mounted using Vectashield (with DAPI [4′,6-diamidino-2-phenylindole]). Parasites that were both red and green were identified as extracellular (attached), whereas those that were green but not red were identified as intracellular (invaded). Images of 15 random fields of view within each well were captured at a magnification of ×600, and the total numbers of intracellular parasites and host cell nuclei were enumerated.

### Attachment assays.

Purified tachyzoites were treated with 3 μM mycalolide B (Enzo Life Sciences) for 10 min, washed three times with DMEM–10% fetal bovine serum (FBS), added onto HFF monolayers in an eight-well chamber slide (2 × 10^6^ parasites per well), and incubated at 37°C for 30 min. Slides were then washed three times to remove unattached parasites, fixed, blocked, and stained with mouse anti-SAG1. The slides were further washed and stained with secondary antibody, namely, Alexa Fluor 594-conjugated goat anti-mouse antibody (Molecular Probes). After 1 h, slides were washed and mounted using Vectashield (with DAPI). Images of 15 random fields of view within each well were captured at a magnification of ×600, and the total numbers of attached parasites and host cell nuclei were enumerated.

### Microneme secretion assays.

Ethanol-induced microneme secretion was performed according to previously described procedures (63). Briefly, freshly egressed parasites were filter purified, washed with DMEM–20 mM HEPES, and resuspended in DMEM–20 mM HEPES (4 × 10^8^ parasites/ml). A 200-μl volume of parasites was then added to 200 μl of preheated (37°C) DMEM–2% ethanol in Eppendorf tubes, and excreted/secreted antigen (ESA) induction was performed for 2 min at 37°C. Tubes were then placed immediately on ice for 5 min, followed by centrifugation at 1,000 × *g* for 10 min at 4°C. A total of 350 μl of the supernatant was removed and centrifuged again, followed by removal of 300 μl and addition of 5× SDS-PAGE sample buffer. The ESA fraction samples were then heated at 100°C for 5 min and resolved on a 4% to 20% gradient gel (Bio-Rad, Hercules, CA). Proteins were transferred from the gel to a nitrocellulose membrane using a wet-transfer apparatus (Bio-Rad, Hercules, CA) at 100 V for 1 h. After blocking with 5% (wt/vol) skim milk powder–Tris-buffered saline (TBS) was performed, membranes were probed with rabbit anti-MIC2 (1:5,000), rabbit anti-M2AP (1:2,000), rabbit anti-PLP1 (1:10,000), rabbit anti-MIC4 (1:2,000), and mouse anti-GRA7 (1:5,000) for 1 h. Membranes were then washed and incubated with either horseradish peroxidase (HRP)-conjugated goat anti-rabbit IgG (Sigma) or HRP-conjugated goat anti-mouse IgG (Sigma). After washing, membranes were treated with SuperSignal West Femto chemiluminescent substrate (Pierce Chemical) and imaged using FluorChem E (ProteinSimple).

### Statistical analysis.

Unless otherwise stated, data were analyzed by one-way analysis of variance (ANOVA) followed by Tukey’s test for multiple comparisons using the GraphPad Prism (v. 7.0c) software package.

### RNA sequencing and differential gene expression analysis.

Total RNA from three independent biological replicates of freshly egressed TgTKL1-HA and TgTKL1-KO parasites was isolated using an RNeasy kit (Qiagen). The quality of total RNA samples was verified by using a BioAnalyzer (Agilent), followed by digestion with DNase I (NEB). rRNA was removed by the use of a Ribo-Zero rRNA removal kit (human/mouse/rat) (Illumina). Sequencing libraries were then generated using a TruSeq RNA Sample Prep kit (v2; Illumina) according to manufacturer’s protocol. Libraries were amplified using a TruSeq cluster kit (v3; Illumina) and subjected to 50-bp single-ended sequencing with an Illumina HiSeq 2000 system.

Data were analyzed using the Bioconductor suite of packages for the R computing environment (v. 3.3.2) and RStudio (v. 1.0.44). Reads were aligned to the *Toxoplasma* GT1 reference genome (ToxoDB v. 29; http://toxodb.org/toxo/) using the Rsubread package (v. 1.26.0) (92), with default settings, followed by featureCounts (93) for gene-level count summarization. Following normalization and filtering, differentially expressed genes were identified by linear modeling and Bayesian statistics using the limma package for R (93). Graphs were generated using Prism (GraphPad, v7.0c) and the ggplot2 package for R (ISBN: 978-0-387-98140-6). For validation of differentially expressed genes, total RNA was isolated from each strain as described above and used to generate cDNA using a QuantiTect reverse transcription kit (Qiagen). Quantitative RT-PCR was performed using Fast SYBR green master mix (Thermo, Fisher), and data were analyzed as described in reference 94 and normalized to alpha-tubulin levels. The primers used for qRT-PCR analysis are listed in Table S3.

### *In vivo* virulence assays.

Six-week-old female CBA/J mice were injected intraperitoneally with TgTKL1-HA, TgTKL1-KO, or TgTKL1-CO parasites (500 parasites in 100 μl PBS; five mice per strain). To verify the viability of the injected parasites, parasites equal in number to those used for the mouse injections and from the same preparation were used to inoculate an HFF monolayer for plaque assays immediately following injection. Seroconversion of all surviving mice was confirmed with Western blot analysis using serum collected 3 weeks postinfection and RHΔ*Ku80* parasite lysate. For rechallenge experiments, five naive 10-week-old female CBA/J mice and the five mice that survived the initial TgTKL1-KO infection were injected with 500 RHΔ*Ku80* tachyzoites (100 μl in PBS). The viability of both of the injected parasite strains was again confirmed using plaque assays as described above.

### Accession number(s).

Accession numbers for data determined in this study are listed in Table 1.
