# Supplementary figures and images for: TgTKL1 Is a Unique Plant-Like Nuclear Kinase That Plays an Essential Role in Acute Toxoplasmosis
Source: mBio. 2018 Mar 20;9(2):e00301-18. doi: 10.1128/mBio.00301-18 (PMC5874906; doi:10.1128/mBio.00301-18)

Figure S1

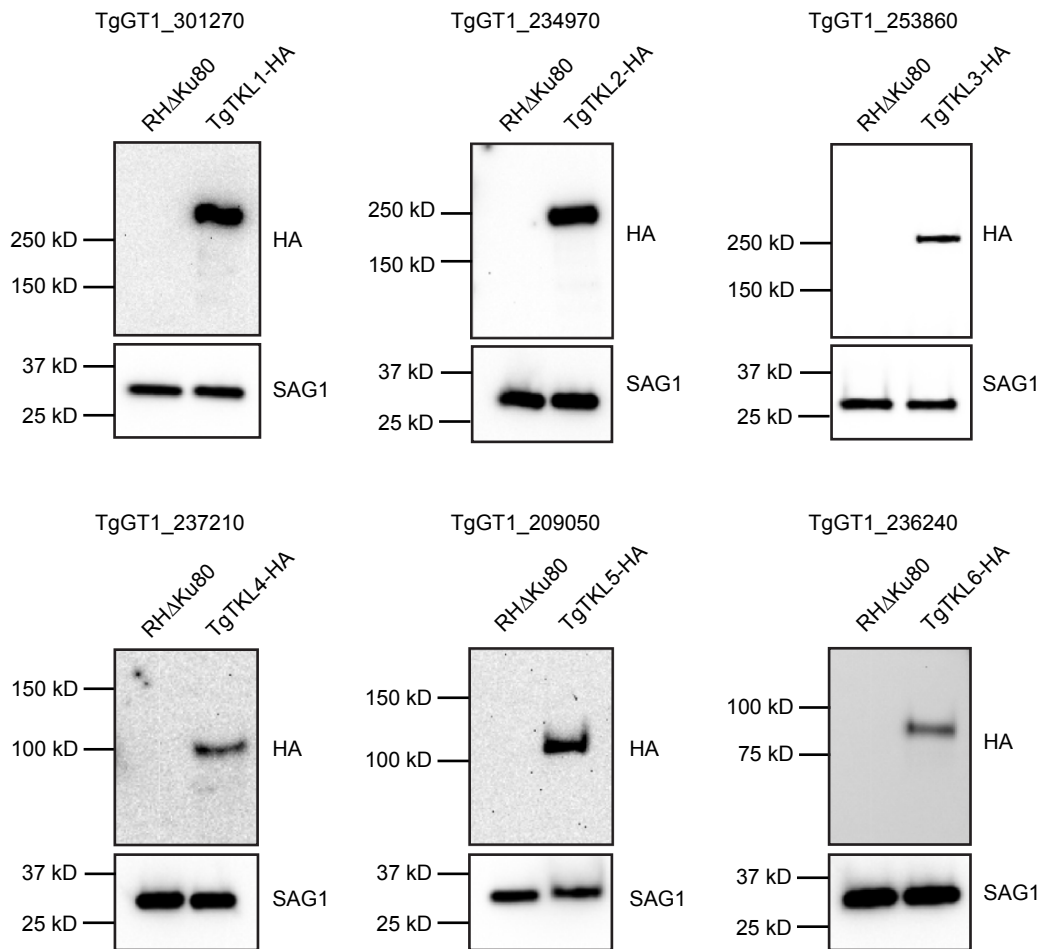

Supplement: FIG S1 [file mbo002183796sf1.pdf]

Figure S2

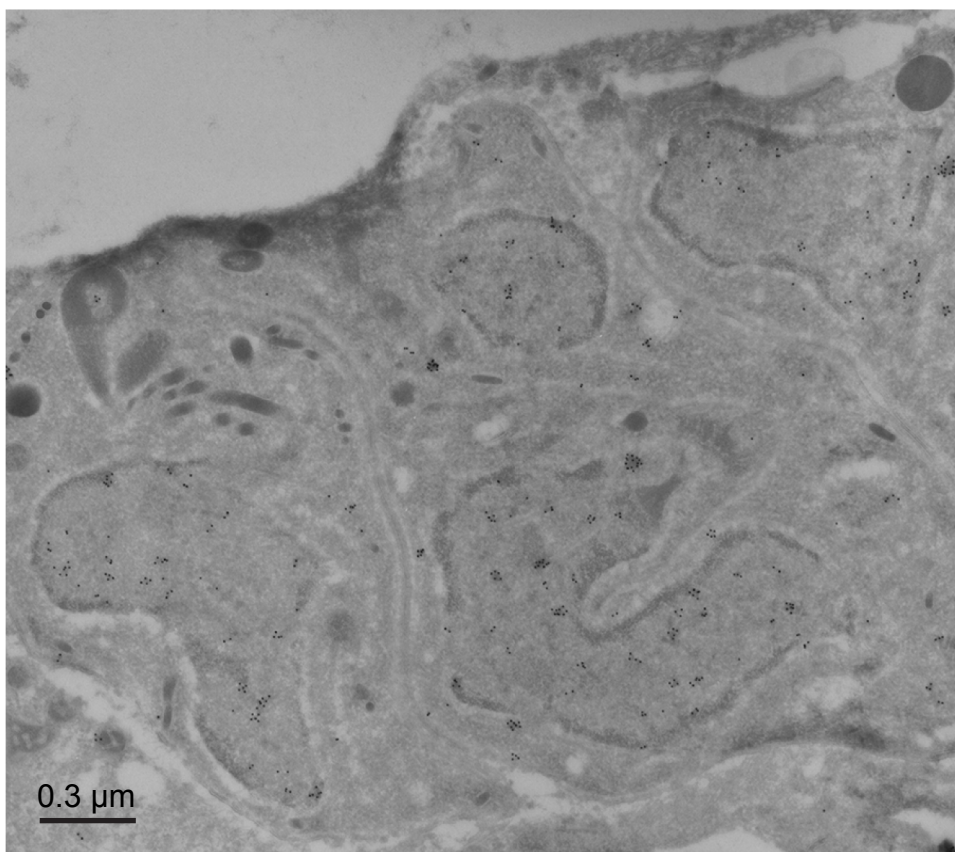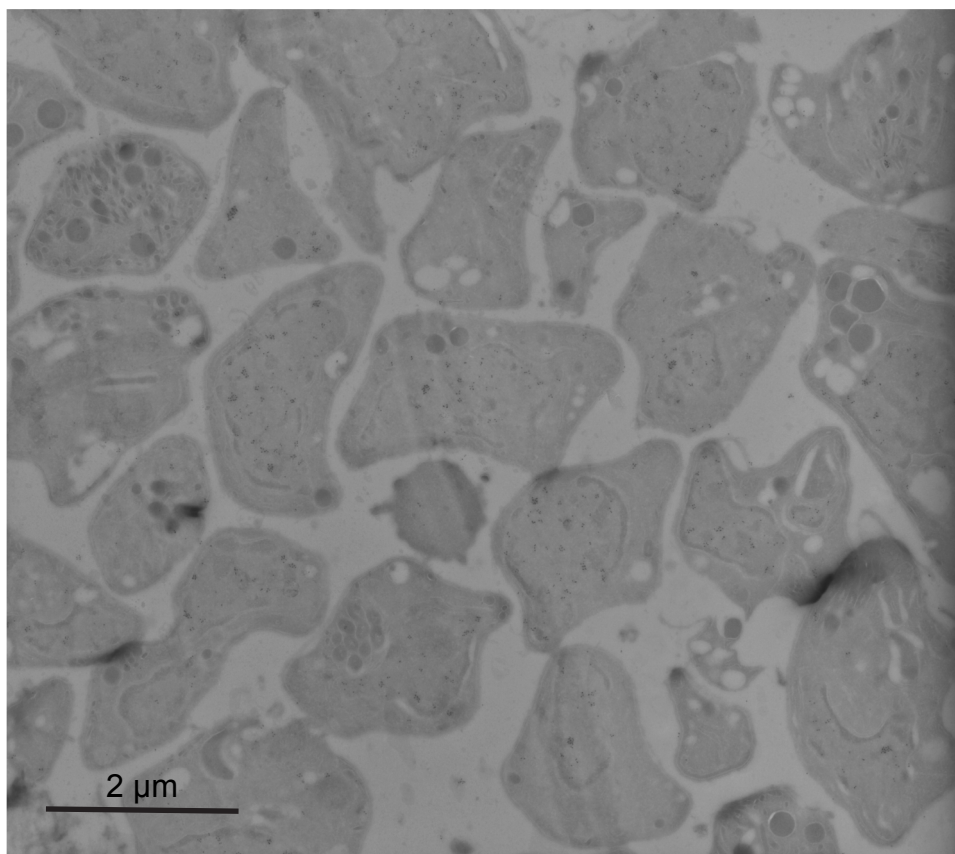

Supplement: FIG S2 [file mbo002183796sf2.pdf]

Figure S3

A

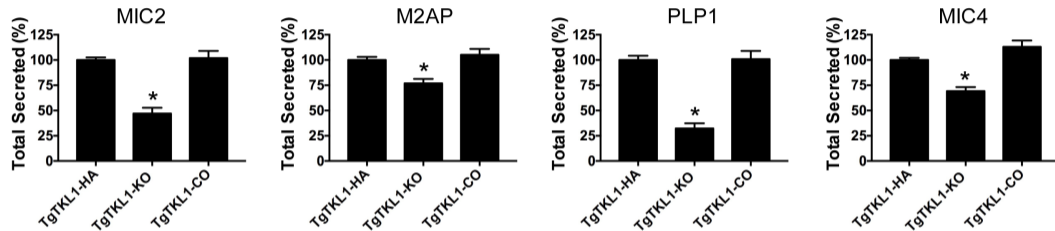

B

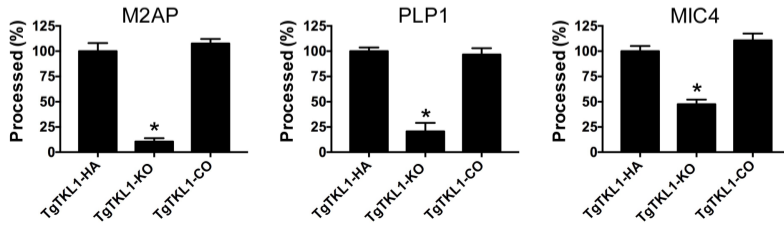

Supplement: FIG S3 [file mbo002183796sf3.pdf]

Figure S4

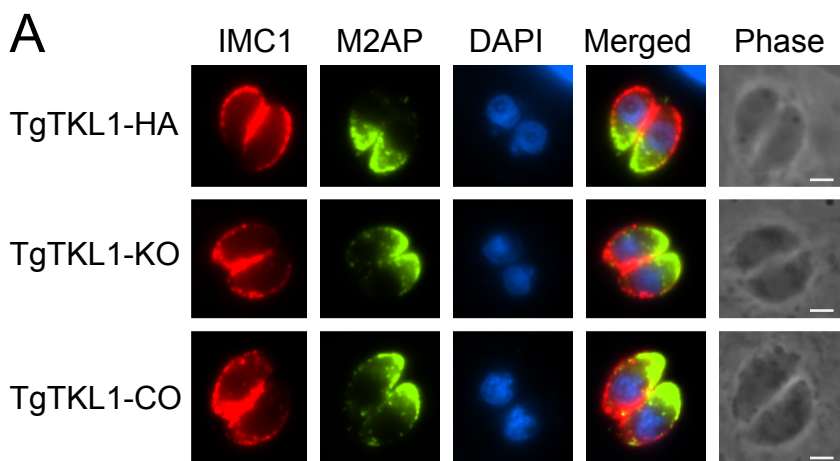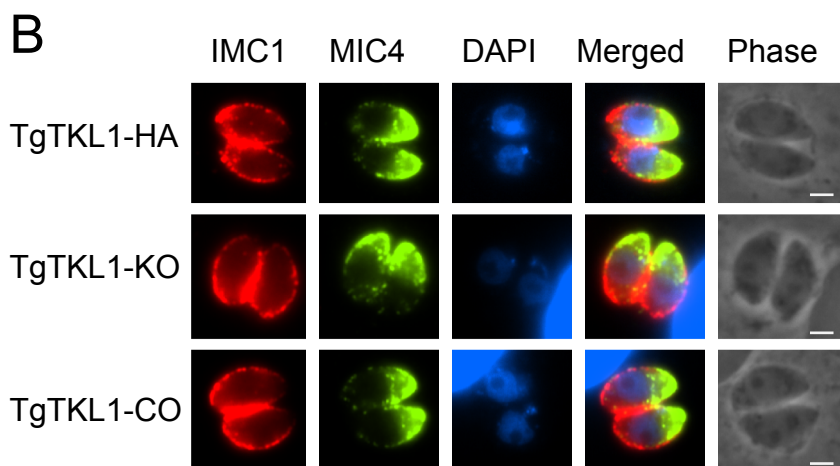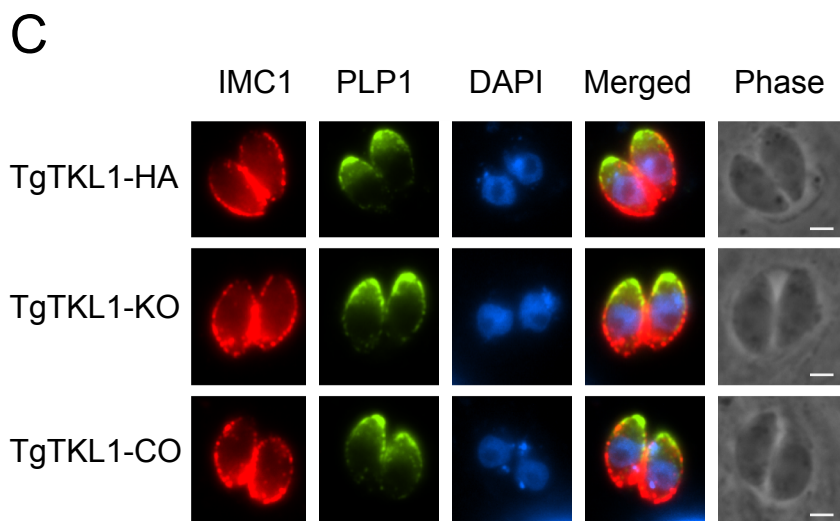

Supplement: FIG S4 [file mbo002183796sf4.pdf]

# Figure S5

## Down-regulated in TgTKL1-KO

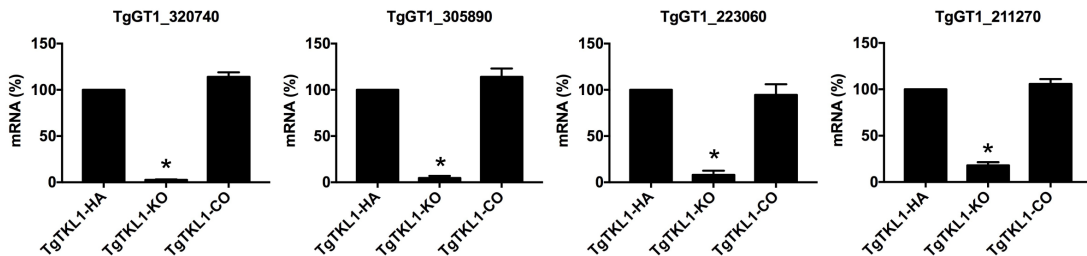

## Up-regulated in TgTKL1-KO

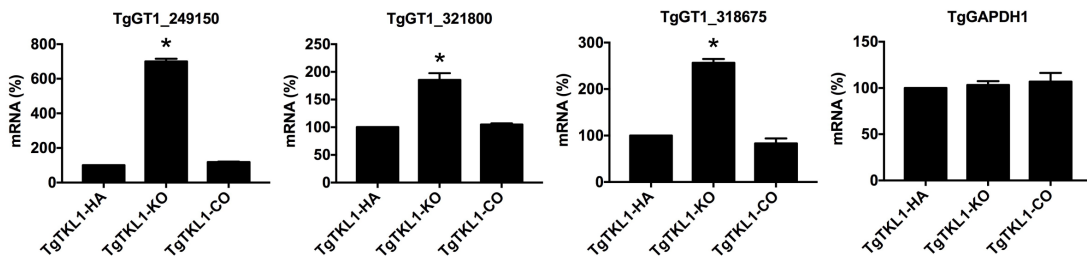

Supplement: FIG S5 [file mbo002183796sf5.pdf]
