# Supplementary material for: TgTKL1 Is a Unique Plant-Like Nuclear Kinase That Plays an Essential Role in Acute Toxoplasmosis
Source: mBio. 2018 Mar 20;9(2):e00301-18. doi: 10.1128/mBio.00301-18 (PMC5874906; doi:10.1128/mBio.00301-18)
Supplement: TABLE S1 [file mbo002183796st1.docx]

| Primer Name | Sequence (5’ to 3’) |
| --- | --- |
| TKL1.HA.F | ttccaatccaatttaattaaGAAGAAGAGCGACGACTGTCTCCGA |
| TKL1.HA.R | ccacttccaattttaattaaACTCAAACTCCCCGACCCT |
| TKL2.HA.F | ttccaatccaatttaattaaGCACGCATCTGTCCATCG |
| TKL2.HA.R | ccacttccaattttaattaaGAGATTAGGTCTTCCTGCAAGACC |
| TKL3.HA.F | ttccaatccaatttaattaaCGCGAAACATTCTCCACAGG |
| TKL3.HA.R | ccacttccaattttaattaaTGCCAAGCCAAAGAAGTCG |
| TKL4.HA.F | ttccaatccaatttaattaaGCTTCTTTGATTCACTTGATGACACGG |
| TKL4.HA.R | ccacttccaattttaattaaCAGTCCGTCGATGATGATCTCC |
| TKL5.HA.F | ttccaatccaatttaattaaGCATCGTCGCGCCAAT |
| TKL5.HA.R | ccacttccaattttaattaaCTTGACTCGTCGTGTGTTCAT |
| TKL6.HA.F | ttccaatccaatttaattaaGCAGATGCTCGGGTGTATTCT |
| TKL6.HA.R | ccacttccaattttaattaaTCTTGGCTGGGCGAAGG |

Table S1. Primers used for tagging TKL genes in *Toxoplasma gondii*
