# Supplementary material for: TgTKL1 Is a Unique Plant-Like Nuclear Kinase That Plays an Essential Role in Acute Toxoplasmosis
Source: mBio. 2018 Mar 20;9(2):e00301-18. doi: 10.1128/mBio.00301-18 (PMC5874906; doi:10.1128/mBio.00301-18)
Supplement: TABLE S2 [file mbo002183796st2.docx]

| Primer Name | Sequence (5’ to 3’) |
| --- | --- |
| 234970.5.KpnI.F | gatcGGTACCCAGCACACGTGGTCACAATCAG |
| 234970.5.HindIII.R | gatcAAGCTTATTTGCGTCTCCTTGTGCCAGC |
| 234970.3.BamHI.F | gatcGGATCCAGGCAGAAAGGCAGTGACAGTG |
| 234970.3.NotI.R | gatcGCGGCCGCTCCGATCTTCTGGAAGTGAGCG |

Table S2. Primers used for generating TKL1 knockout strain.
