# Supplementary material for: TgTKL1 Is a Unique Plant-Like Nuclear Kinase That Plays an Essential Role in Acute Toxoplasmosis
Source: mBio. 2018 Mar 20;9(2):e00301-18. doi: 10.1128/mBio.00301-18 (PMC5874906; doi:10.1128/mBio.00301-18)
Supplement: TABLE S3 [file mbo002183796st3.docx]

| Primer Name | Sequence (5’ to 3’) |
| --- | --- |
| 320740.F | GTGGTCTCAGATTTCCTGCTCT |
| 320740.R | TGATCGAGTCGAACCAGACGTC |
| 305890.F | ACGATCTTCACCAAGCATGAGG |
| 305890.R | AAGAAGCTCCACCAGAGCCATG |
| 223060.F | CATGCACAGAATGTGCCTTGGC |
| 223060.R | CAGGTTCCACGCTGCAGTTGAC |
| 211270.F | GCCGTTGACGTATATCGACTTG |
| 211270.R | CTTCTCCCAGTCCTACGGCATG |
| 249150.F | TGTCGGTAGCGAGATGACAGAG |
| 249150.R | CAATCCATCAGTCGCCAACACC |
| 321800.F | CTCAATCAGGGAGCACCACATG |
| 321800.R | GGTTCGCACAGGTCGTTCGTTG |
| 318675.F | TGACGCAGATGAGGATGCTGAC |
| 318675.R | TTGTTGCCTCCGTTTCCTGCGC |
| 301270.F | TCCACTCGGGAGAAGAGAGA |
| 301270.R | AACGAGCTTCAGCGCATATT |
| TKL1.F | AGCTTAGAAGCCGTCACCAA |
| TKL1.R | GAGGCCTCGTGAAGTCAGTC |
| TUBA1.F | GCATGATCAGCAACAGCACT |
| TUBA1.R | GAGAGCAGCCAAATCCTCAC |

Table S3. Primers used for qRT-PCR analysis.
